# Tumor senescence leads to poor survival and therapeutic resistance in human breast cancer

**DOI:** 10.3389/fonc.2023.1097513

**Published:** 2023-03-02

**Authors:** Jingtong Zhai, Jiashu Han, Cong Li, Dan Lv, Fei Ma, Binghe Xu

**Affiliations:** ^1^ Department of Medical Oncology, National Cancer Center/National Clinical Research Center for Cancer/Cancer Hospital, Chinese Academy of Medical Sciences and Peking Union Medical College, Beijing, China; ^2^ 4 + 4 Medical Doctor Program, Chinese Academy of Medical Sciences & Peking Union Medical College, Beijing, China

**Keywords:** breast cancer, senescence, tumor microenvironment, prognosis, cancer therapy

## Abstract

**Background:**

Breast cancer (BRCA) is the most common malignant tumor that seriously threatens the health of women worldwide. Senescence has been suggested as a pivotal player in the onset and progression of tumors as well as the process of treatment resistance. However, the role of senescence in BRCA remains unelucidated.

**Methods:**

The clinical and transcriptomic data of 2994 patients with BRCA were obtained from The Cancer Genome Atlas and the METABRIC databases. Consensus clustering revealed senescence-associated subtypes of BRCA patients. Functional enrichment analysis explored biological effect of senescence. We then applied weighted gene co-expression network analysis (WGCNA) and LASSO regression to construct a senescence scoring model, Sindex. Survival analysis validated the effectiveness of Sindex to predict the overall survival (OS) of patients with BRCA. A nomogram was constructed by multivariate Cox regression. We used Oncopredict algorithm and real-world data from clinical trials to explore the value of Sindex in predicting response to cancer therapy.

**Results:**

We identified two distinct senescence-associated subtypes, noted low senescence CC1 and high senescence CC2. Survival analysis revealed worse OS associated with high senescence, which was also validated with patient samples from the National Cancer Center in China. Further analysis revealed extensively cell division and suppression of extracellular matrix process, along with lower stromal and immune scores in the high senescence CC2. We then constructed a 37 signature gene scoring model, Sindex, with robust predictive capability in patients with BRCA, especially for long time OS beyond 10 years. We demonstrated that the Sene-high subtype was resistant to CDK inhibitors but sensitive to proteosome inhibitors, and there was no significant difference in paclitaxel chemotherapy and immunotherapy between patients with different senescence statuses.

**Conclusions:**

We reported senescence as a previously uncharacterized hallmark of BRCA that impacts patient outcomes and therapeutic response. Our analysis demonstrated that the Sindex can be used to identify not only patients at different risk levels for the OS but also patients who would benefit from some cancer therapeutic drugs.

## Introduction

Breast cancer (BRCA) is the most common malignant tumor and the leading cause of cancer-related death in women, making it a serious threat to human health (1). Due to the multifaceted nature of this complex disease, patients with BRCA are often classified into luminal A, luminal B, human epidermal growth factor receptor 2 (HER2)-enriched, and basal-like subtypes based on molecular expression by pathology (2, 3). Moreover, the Tumor, Nodes, Metastases (TNM) staging system, based on anatomical abnormalities including tumor size, lymph node involvement, and distant metastatic status, is the most frequently used tool for outcome prediction (4). However, there is still considerable heterogeneity in the treatment responses and the clinical outcomes among patients with similar clinical and pathologic backgrounds (5), highlighting the need for novel predictive markers other than clinical stages and pathohistological classifications.

As next-generation sequencing technologies can provide more information at a now acceptable cost, classification of patients with BRCA based on transcriptomic profiles has come to use, including the Prediction Analysis of Microarray 50 (PAM50), the 21-gene assay (OncotypeDX) and the 70-gene signature (MammaPrint) (6–8). Beside these classification systems based on the whole expression profile, there are also several signatures based on key biological features of the tumor, such as autophagy-related signatures (9, 10), N6-methyladenosine (11, 12), immune cell infiltration (13, 14), etc. These feature-specific signatures bring forth the hope for precision medicine, but many important biological processes remain uninvestigated.

Cellular senescence is the growth arrest of cells that have been intrinsically and/or extrinsically damaged by factors including oncogenic activation, mitochondrial dysfunction, radiation damage, oxidative and genotoxic stress, and chemotherapeutic agent-induced damage (15). Accumulating evidence suggests that cellular senescence is a double-edged sword in human cancer. On one hand, cellular senescence is considered tumor-suppressive by inhibition of cell division and tumor expansion (16–18). On the other hand, senescence-associated cellular plasticity and stemness reprogramming may be critical for treatment-resistance in many cancer types, including BRCA (19–21). Moreover, secretion of senescent cells (inflammatory cytokines, chemokines, growth factors), known as the senescence-associated secretory phenotype (SASP), influences cancerous, stromal, and immune cells in the tumor microenvironment (TME) (22–24). Despite numerous preclinical studies in cellular and animal models, our understanding on the variable effects of senescence in different cancers is still rudimentary, and the characteristics of senescence in patients with BRCA are particularly complex (25–27). Therefore, it is essential to elucidate the value of tumor senescence as a biomarker to guide clinical prognosis and treatment for better cancer prevention and therapy.

**Figure 1:**
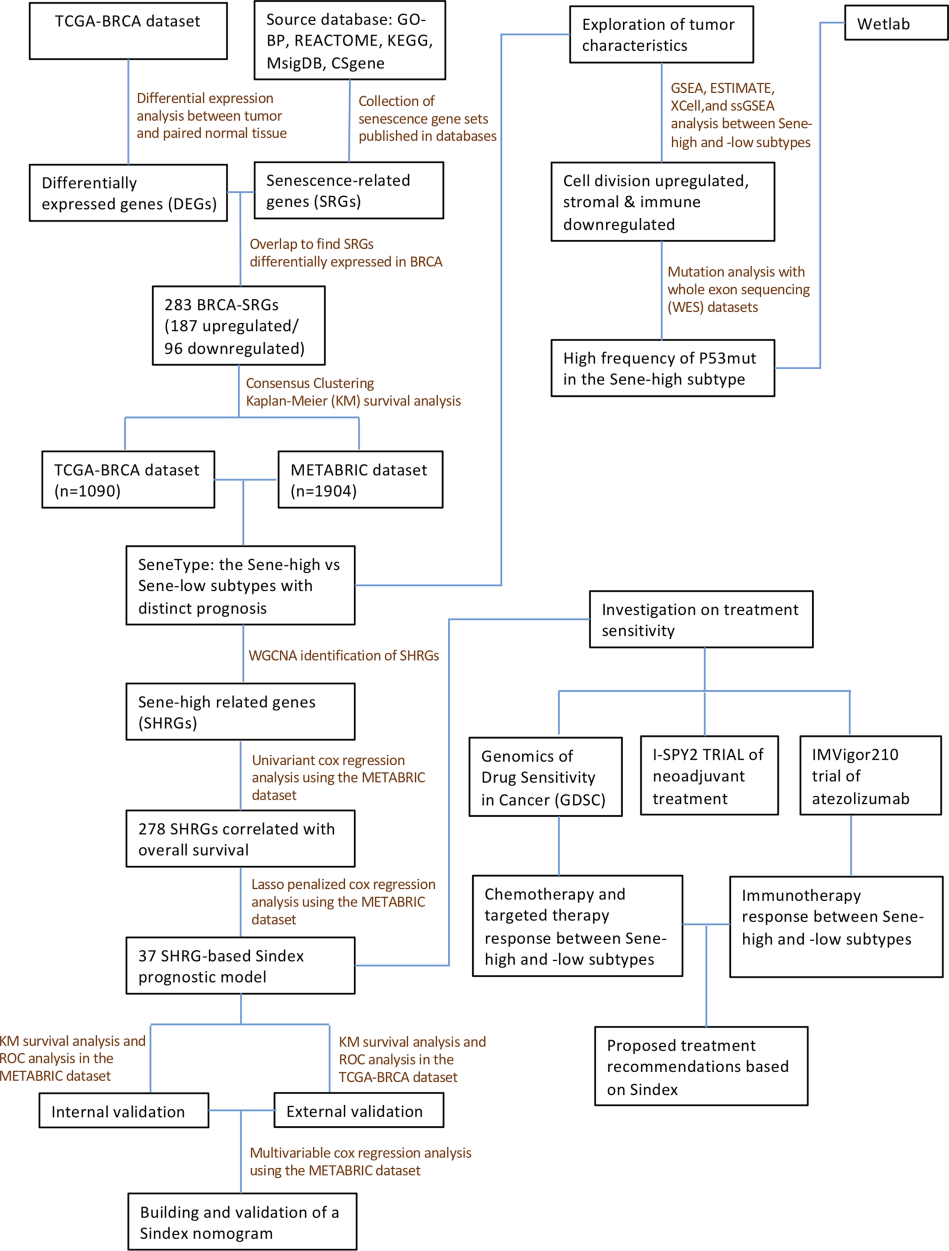
The flow chart describing the number of cases and workflow included in the study cohort.

In this study, we comprehensively analyzed the senescent features in multiple BRCA cohorts and classified BRCA patients into two subtypes with distinct senescent status, microenvironment composition, mutation frequency, and signaling pathway activation. We also constructed the Senescence Index (Sindex) model, a tumor senescence scoring system that not only predicted the survival outcome of patients with BRCA, but also suggested potentially effective treatment strategies for the high-risk, high-senescence group of patients (**Figure 1**).

## Materials and methods

### Data collection and processing

We acquired publicly available datasets and information of patients with BRCA from The Cancer Genome Atlas (TCGA, https://portal.gdc.cancer.gov/) and the Molecular Taxonomy of Breast Cancer International Consortium (METABRIC) databases (cBioPortal, https://www.cbioportal.org/). For the TCGA dataset, raw count expression data was downloaded from the TCGA Data Portal with the TCGAbiolinks R package (28), and then the raw counts were transformed into normalized count with the VST function from the Deseq2 R package for subsequent analysis (29). Microarray gene-expression data was downloaded from the METABRIC database (https://www.cbioportal.org/study/summary?id=brca_metabric). The resources used in this study were summarized in **Supplementary Table 4**. All the R packages used in this study run in the R software (version 4.1.2, https://www.r-project.org). Data from the clinical trial I-SPY2 and IMvigor 210 was deposited under GSE196096 and GSE145281 (30, 31).

### Patient population and clinical information

In this study, we retrospectively collected 2994 patients with BRCA for subsequent analysis: the METABRIC cohort and TCGA cohort each included 1904 and 1090 patients with detailed clinical information, survival follow-up, and complete genomic data, respectively (**Supplementary Table 5**). In addition, we collected pathological tissue sections and clinical data of patients with BRCA from the National Cancer Center of China. This study was approved by the ethics committee of the Chinese Academy of Medical Sciences and Peking Union Medical College (Ref. 19/327-2111).

### Identification of differentially expressed genes and GSEA analysis

The DEGs between BRCA and normal breast tissues were identified with the “Deseq2” R package based on raw count data from the TCGA dataset (29). Genes with a false discovery rate < 0.05 and the |fold change| > 1 were defined as significant DEGs. Gene set enrichment analysis (GSEA) was applied to elucidate biological differences between BRCA and normal adjacent tissue (NAT) (32).

### Consensus clustering for the senescence subtypes

Based on BRCA-senescence related genes, patients with BRCA were clustered with “ConsensusClustering Plus” R package (33). Single-sample GSEA (ssGSEA) was performed for senescence related Reactome gene sets using “GSVA” R package (34), and the results were visualized with the R package “ComplexHeatmap” (35). Principal component analysis (PCA) plot was visualized using “PlotPCA” function of the Deseq2 R package (29).

### Characterize biological effects of senescence

We first used GSEA to compare difference in biological pathway activities between CC1 and CC2; then calculated the activity of hallmark pathways with ssGSEA, and investigated each pathway’s correlation with senescence. The tumor purity scores, immune infiltration levels, and stromal contents in different samples were evaluated *via* the “ESTIMATE” algorithm (36). To evaluate stromal and immune cell infiltration characteristics of BRCA, we used the “Xcell” R package (37) to quantitatively analyze the infiltration levels of different immune cells and fibroblasts. The ssGSEA algorithm was used to quantify the activity of immunogenic cell death, apoptosis, TGF-b, extracellular matrix (ECM) assembly, and angiogenesis, all gene sets retrieved from GO-BP. For WES data, the R package “Maftools” was utilized to calculate tumor mutation burden (TMB) and compare mutation differences between patients with high and low senescent statuses (38).

### Multiplex immunofluorescence immunohistochemistry and imaging

A tissue microarray (TMA) spotted with tumor samples from 74 BRCA patients (HBreD090PG01) was purchased from Shanghai Outdo Biotech Co. Ltd. A total of 90 cores on the slide consisted of 74 cases of BRCA tissue and paired normal breast tissue. All tissues were collected in accordance with the ethical standards with the donor being informed completely and with their consent, from National Human Genetic Resources Sharing Service Platform: 2005DKA21300. For multiplex immunofluorescence staining, the manual multiplex immunofluorescence immunohistochemistry kit was used according to the manufacturer’s instructions, and the molecules panel, which consisted of five antibodies including anti-PANCK, anti-α-SMA, anti-CD8, anti-CD68, anti-P16, was conducted on the same slide.

### WGCNA and Sindex construction and validation

All model construction work was first established in the METABRIC dataset, and then tested in TCGA as external validation. We first used the “WGCNA” package in R (with default parameters) to identify gene modules associated with high senescence, defining the module with the highest absolute module significance as the key module (red). To develop a senescence-based signature of individual tumor with better clinical utility and practicability, we first used the “Ezcox” R package to apply univariate proportional hazards (Cox) regression analysis to preliminarily screen overall survival (OS)-related genes in the module. Then, to remove the multicollinearity among these genes, we applied least absolute shrinkage and selection operator (LASSO) regression with the optimal penalty parameter and a minimum 10-fold cross-validation to identify the most valuable prognostic genes (39). The final 37 genes were used to establish a final model with Cox regression, and the linear predictor values were defined as the Sindex, based on which survival probabilities were calculated with the equation from Cox regression. Time-dependent receiver operating characteristic (ROC) curves were made using survivalROC R package, conducting calibration at different timepoints to determine the robustness of the model. Moreover, the Sindex of patients were used to divided patients into high and low subgroups according to an optimal cutoff value selected by the R package “maxstat” (40), and generated survival curves *via* the Kaplan-Meier (K-M) method and log-rank test with the R packages “survival” and “survminer” (41). Collectively, area under the curve (AUC) of the ROC curve and K-M survival analysis were considered when evaluating the predictive ability of the model.

For nomogram construction, we first used univariant Cox regression to identify survival-related clinical characteristics (including Sindex). The independent predictive characteristics were screened out with multivariable Cox regression model, and visualized with a forest plot drawn by the survminer R package. The stepwise multivariate Cox regression was used to construct the final nomogram, which included lymph nodes, tumor size, Nottingham prognostic index, inferred menopausal state, age and Sindex. The nomogram was further evaluated for predictive probability of 5-, 10-, and 15-year survival in patients with BRCA using the “survival” R package (41).

### Exploration of the significance of senescore in response to cancer therapy

To evaluate the potential value of Sindex in therapeutic suggestion for patients with BRCA, we calculated the half-maximal inhibitory concentration (IC50) of common therapeutic drugs based on the Genomics of Drug Sensitivity in Cancer (GDSC) databases through the Oncopredict algorithm (42). The IC50 of these chemotherapeutic drugs in high and low Sindex subgroups were compared by Wilcoxon test with the results exhibited in box diagrams using the “ggpubr” R package (43). I-SPY2 is an ongoing multicenter, phase II neoadjuvant platform trial for patients with BRCA that used a variety of treatments and combinations (30). Based on the clinical response and transcriptomic data, we calculated Sindex for each patient, and compared the average Sindex between pathologic complete response (pCR) and non-pCR patients treated with each treatment schedule. IMvigor210 is a phase III clinical trial of atezolizumab in urothelial cancer patients. We calculated the Sindex for each patient and compared the average Sindex between responders (R) [complete response (CR), partial response (PR)] and non-responders (NR) [stable disease (SD), progressive disease (PD)] to elucidate the effect of senescence on immunotherapy response.

### Cell lines and reagents

In this study, we used the breast cancer cell line MDA-MB-436 obtained from ATCC and pancreatic cancer cell line SW1990 with P53 knockout kindly provided by Prof Ziwen Liu. Both cell lines were grown in the growth medium recommended by ATCC, Leibovitz’s L-15 medium (Thermo Fisher, 11415064) with1% penicillin and streptomycin (Thermo Fisher, 15070063), and 10% and 20% fetal bovine serum (Thermo Fisher, 10099), respectively. Cell cultures are regularly tested for mycoplasma infection with MycoAlert mycoplasma detection kits (Lonza, LT07).

### Cloning of TP53 vectors

Wildtype and mutant TP53 vectors were amplified from SW1990 (TP53WT) and PANC1 (TP53MUT, c.818G>A, p.R273H) cells, respectively, as previously described by Y Liu and W Bodmer (https://www.ncbi.nlm.nih.gov/pmc/articles/PMC1327731/). The TP53 vectors are then cloned into pInducer20 (Addgene) through the Gateway method: briefly, 50 ng of pCR8 TP53 and 150 ng of pInducer20 plasmids (Addgene) were mixed with LR Clonase II (Invitrogen, 11791) and incubated at 25 °C for 1 hour before reaction termination by Proteinase K. The reaction products were then used to transform DH5a bacteria and positive clones were selected on ampicillin agar plate.

### P53 re-expression

Cell lines with stable overexpression of p53 WT or MUT were generated with lentivirus transduction. Lentiviruses were produced by transfecting Lenti-HEK-293 cells with 2 μg of p53 construct, 1.5 μg of viral protein R (VPR), and 0.5 μg of vesicular stomatitis Indiana virus G protein (VSVG), using Lipofectamine 3000 transfection reagent (Thermo Fisher, L3000015). Viral particles were collected from the medium supernatant after centrifuging at 4000 rpm for 30 min. 48 h after infection with 1ml of lentivirus and 10 μg of Polybrene, puromycin was added to select and generate cell lines with stable expression. Stable cells were tested for expression of P53 WT or MUT through sequencing. Transient re-expression was achieved simply through Lipofectamine 3000 aided plasmid transfection according to the manufacturer’s instruction.

### Quantification of mRNA expression

Total RNA was obtained with RNeasy kit (Qiagene 74004) following manufacturer’s instruction. cDNA was synthesized with the M-MLV reverse transcriptase kit (Thermo Fisher, 28025013). RT-qPCR reaction was performed in a final volume of 20 μ containing 12 μl TaqMan^®^ Universal PCR Master Mix, 5 μl H2O, 1 μl of forward and reverse primers, and 1 μl cDNA (approximately 10ng/μl). The reaction was put in an ABI PRISM^®^ 96-Well Optical Reaction Plate under the standard thermal cycling conditions by ABI PRISM^®^ 7000 Sequence Detection System (TaqMan^®^): initial 50°C for 2 min and 95°C for 10 min followed by 40 cycles at 95°C for 15 sec and 60°C for 1 min were used. All reactions were performed in three duplicates. The primers used for qPCR reactions are shown in **Table 1**.

**Table 1:** The primers used for qPCR reactions.

Gene	Forward sequence	Reverse sequence
P53	CCTCAGCATCTTATCCGAGTGG	TGGATGGTGGTACAGTCAGAGC
p16INK4A	CTCGTGCTGATGCTACTGAGGA	GGTCGGCGCAGTTGGGCTCC
P21	AGGTGGACCTGGAGACTCTCAG	TCCTCTTGGAGAAGATCAGCCG
Rb1	CAGAAGGTCTGCCAACACCAAC	TTGAGCACACGGTCGCTGTTAC
IL-1a	TGTATGTGACTGCCCAAGATGAAG	AGAGGAGGTTGGTCTCACTACC
IL-6	AGACAGCCACTCACCTCTTCAG	TTCTGCCAGTGCCTCTTTGCTG
TGFb	TACCTGAACCCGTGTTGCTCTC	GTTGCTGAGGTATCGCCAGGAAA
ICAM1	AGCGGCTGACGTGTGCAGTAAT	TCTGAGACCTCTGGCTTCGTCA

### mIHC staining

Microarray tissue samples collected from breast cancer patients were used as experimental samples and tonsil tissues were used as controls (both as FFPE samples). All the tissues were cut and made as section slides with 2-μm thicknesses. The slides were deparaffinized in xylene for 10 mins and repeat three times, and rehydrated in absolute ethyl alcohol for 5 mins and repeat twice, 95% ethyl alcohol for 5 mins, 75% ethyl alcohol for 2 mins, sequentially. Then the slides were washed with distilled water 3 times. A microwave-oven is used for heat-induced epitope retrieval; during epitope retrieval, the slides were immersed in boiling EDTA buffer (Alpha X Bio, Beijing, China) for 15mins. Antibody Diluent/Block (Alpha X Bio, Beijing, China) was used for blocking. The mIHC staining part was performed and analyzed according to a 6-plex-7-color panel, and specifications (with primary antibodies used) are as the following: CD8 (ZA0508, ZSGB-BIO, CHINA), CD68 (ZM0060, ZSGB-BIO,CHINA), PANCK (ZM0069, ZSGB-BIO,CHINA), P16 (ab51243, Abcam, Cambridge, UK), a-SMA (ab7817, Abcam, Cambridge, UK) and CD31 (ab76533, Abcam, Cambridge, UK). All the primary antibodies were incubated for 1 hr at 37°C. Then slides were incubated with Alpha X Ploymer HRP Ms+Rb (Alpha X Bio, Beijing, China) for 10 mins at 37°C. Alpha X 7-Color IHC Kit (Alpha X Bio, Beijing, China) was used for visualization. The correspondences between primary antibodies and fluorophores are listed in below:

AlphaTSA 480 (CD8), AlphaTSA 520 (CD68), AlphaTSA 570 (PANCK), AlphaTSA 620 (P16), AlphaTSA 690 (CD31) AlphaTSA 780 (a-SMA). After each cycle of staining, heat-induced epitope retrieval was performed to remove all the antibodies including primary antibodies and secondary antibodies. The slides were counter-stained with DAPI for 5 mins and enclosed in Antifade Mounting Medium (I0052; NobleRyder, Beijing, China). Axioscan7 (ZEISS, Germany) was used for imaging the visual capturing. Data analysis was performed with Halo (3.4, Indica Labs, United States).

### Statistics

Statistical analysis was performed based on R software v4.1.2 (https://www.r-project.org/) and GraphPad Prism v9.3.0 (https://www.graphpad.com/). Categorical variables were analyzed using χ2 test or Fisher’s exact test. Continuous variables for paired samples were analyzed using Student’s t test. Multiple groups of continuous variables were analyzed using one-way ANOVA. Survival analysis was performed based on the univariate and multivariate Cox regression. Pearson coefficient of correlation was calculated to measure the correlation between two variables. Unless stated otherwise, two-tailed p < 0.05 was regarded as statistically significant.

## Results

### Senescence-based subtyping of BRCA

**Figure 2:**
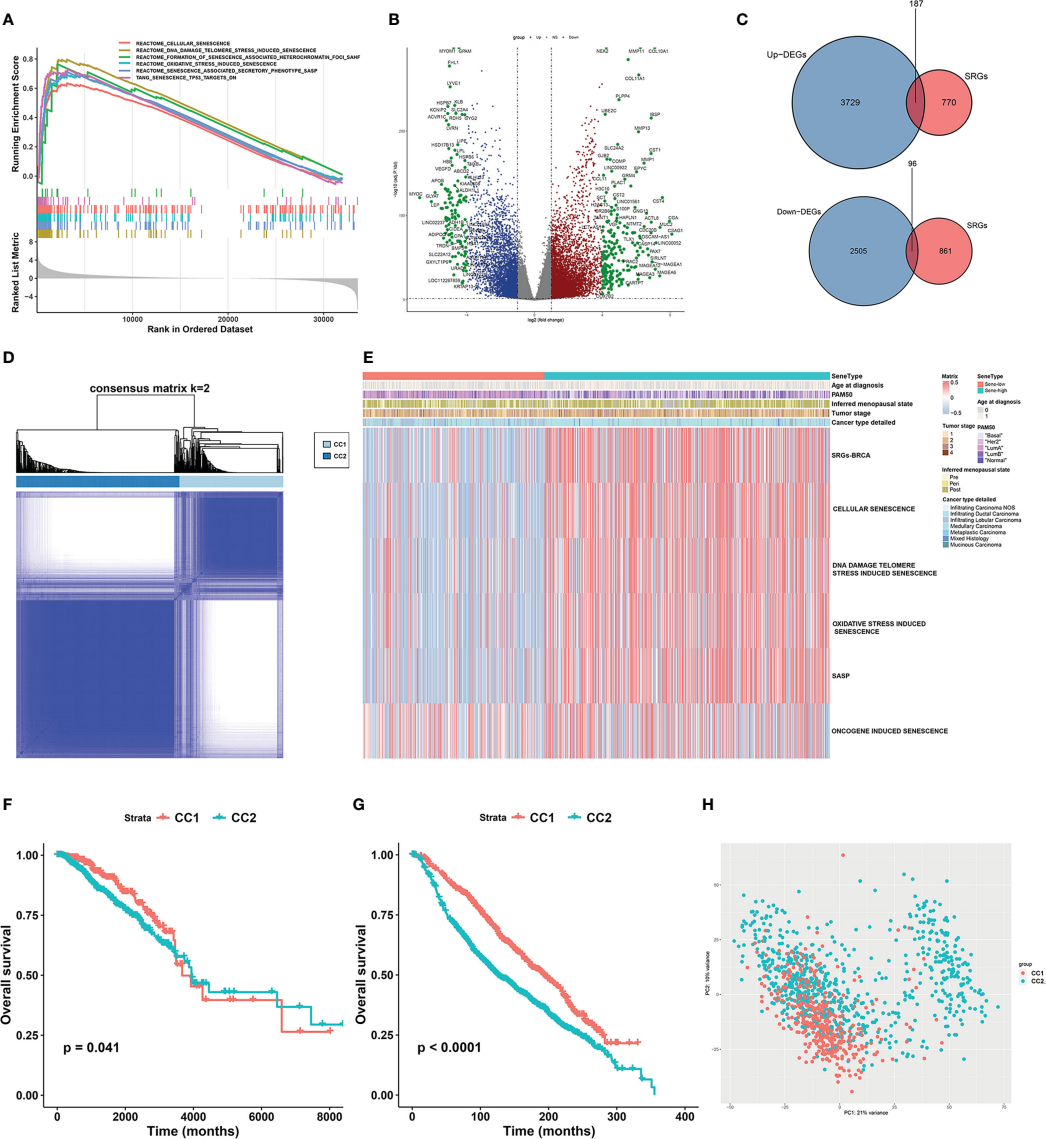
Unsupervised consensus clustering based on senescence-related genes reveals distinct breast cancer subtypes. **(A)** GSEA analysis showed that senescence-related pathways were significantly activated in breast cancer tissues compared to normal tissues in the TCGA-BRCA cohort. **(B)** Volcano plot showing up- and down-regulated differentially expressed genes (DEGs) between breast cancer and normal breast tissue in the TCGA-BRCA cohort. Genes marked in red were up-regulated in breast cancer tissues, and genes marked in blue were down-regulated. **(C)** The Venn diagram showed that the differentially expressed senescence-related genes (SRGs) in breast cancer were obtained by intersecting the results of differential gene analysis of breast cancer with the SRGs gene set. **(D)** Consensus clustering matrix based on differentially expressed SRGs with k=2 in the TGGA-BRCA cohort. **(E)** GSVA enrichment analysis revealed distinct senescence signatures between CC1 and CC2 subtypes in the TCGA-BRCA cohort. Heatmap showing differences in ssGSEA scores for each aging-related gene set between CC1 and CC2 subtypes. **(F, G)** Survival analysis of the two subtypes in the TCGA-BRCA cohort **(F)** and the METABRIC cohort **(G)**. Kaplan-Meier curves showed significant survival differences between CC1 and CC2 subtypes in both cohorts. P-values were calculated by log-rank test. **(H)** Principal component analysis of expression profiles to differentiate the two subtypes in the TCGA-BRCA cohort. CC1 was marked in red and CC2 was marked in green.

We first compared the transcriptomic data of BRCA and paired NAT from the TCGA dataset. GSEA showed extensive cellular senescence in BRCA but not NAT, in terms of biological pathways including cellular senescence (NES = 2.1709, P < 0.001), DNA damage/telomere stress induced senescence (NES = 2.42879, P < 0.001), formation of senescence associated heterochromatin foci (NES = 1.759044, P < 0.001), oxidative stress induced senescence (NES = 2.244097, P < 0.001), senescence associated secretory phenotype (NES = 2.274088, P < 0.001) and senescence TP53 targets DN (NES = 2.123696, P < 0.001) (**Figure 2A**).

To further investigate the role of senescence in breast cancer, we reviewed available literatures and databases for senescence-related genes (SRGs) published in the Kyoto Encyclopedia of Genes and Genomes database, the Gene Ontology database, the Reactome database, the Molecular Signatures Database and the Csgene database. Collectively, we found 28 gene sets and 957 SRGs (**Supplementary Table 1**). We then used Deseq2 to obtain 6236 DEGs between BRCA and NAT, among which 3729 were upregulated and 2505 were downregulated (**Figure 2B**). To identify SRGs with key biological roles in breast cancer, we overlapped the DEGs and the SRGs to get 187 upregulated and 96 downregulated genes, making up the list of 283 BRCA-SRGs (**Figure 2C**).

We then investigated the value of senescence in breast cancer subtyping. Consensus clustering of the TCGA dataset based on the expression of BRCA-SRGs classified patients into two distinct senescence-associated clusters, noted CC1 and CC2 respectively (**Figure 2D**). ssGSEA analysis of widely used Reactome senescence-related gene sets suggested that senescence is activated in CC2 but suppressed in CC1, except for SASP (**Figure 2E**). We further investigated the role of other patient parameters in the above subtyping scheme, and found no differential distribution of age, menopausal stage, tumor stage, and pathological subtype between the two subtypes. Since senescence is often considered an age-related process, we further confirmed that there was no correlation between senescence and patient age (**Supplementary Figure 1A**). However, the PAM50 subtypes were unevenly distributed, with more Luminal B, HER2-enriched, and triple-negative subtypes in CC2, and predominantly Luminal A in CC1 (**Figure 2E**, **Supplementary Figure 1B**). Similar results were obtained from the METABRIC dataset (**Supplementary Figures 1C–F**).

Survival analysis between CC1 and CC2 revealed worse OS associated with CC2 in both TCGA and METABRIC (**Figures 2F, G**). Moreover, PCA based on transcriptomic data revealed clear separation, suggesting distinct molecular characteristics and biological behaviors between the two subtypes (**Figure 2H**, **Supplementary Figure 1G**).

### Biological effect of senescence

**Figure 3:**
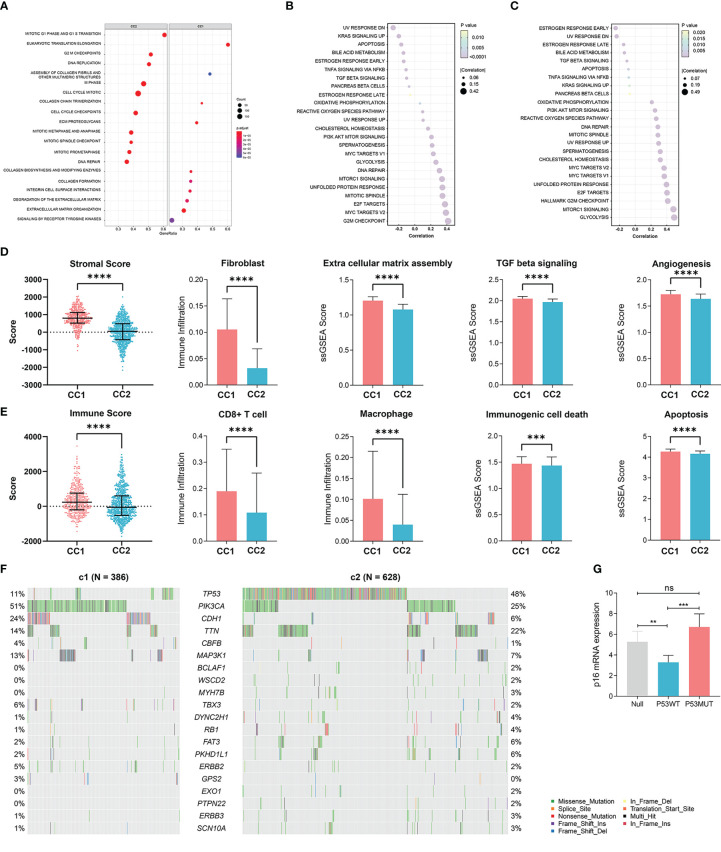
Elucidation of the biological impacts of senescence on the tumor biological process and the tumor microenvironment. **(A)** GSEA enrichment showing upregulated pathways in CC2 versus CC1. **(B, C)** The correlation between senescence and hallmark pathways of cancer in **(B)** TCGA-BRCA dataset and **(C)** the Metabric adtaset. **(D)** Comparison of stromal condition between CC1 and CC2, including stromal score calculated by Estimate, fibroblast cell number calculated by X cell, extracellular matrix assembly, TGF beta signaling, angiogenesis score calculated by ssGSEA. **(E)** Comparison of immune compartment between CC1 and CC2, including immune score calculated by Estimate, numbers of CD8^+^ T cells and macrophages calculated by Xcell, immunogenic cell death and apoptosis calculated by ssGSEA. **(F)** Comparison of mutation frequencies between CC1 and CC2 in the TCGA-BRCA dataset. **(G)** The effect of wildtype P53 (P53WT) and mutant P53 (P53MUT) re-expression on senescence in terms of p16 mRNA content measure by RT-qPCR in the P53 truncated cell line MDA-MB-436 **p < 0.01, ***p < 0.001, ****p < 0.0001. ns, no significance.

We next elucidated the impact of senescence on the behaviors of BRCA, through which high senescence mediated poor survival. We performed GSEA enrichment analysis between CC1 and CC2 in both METABRIC (**Figure 3A**) and TCGA (**Supplementary Figure 2A**), revealing extensively cell division and suppression of ECM process in CC2. As to the hallmark characteristics of tumor, correlation analysis between senescence and hallmark pathways in both METABRIC (**Figure 3B**) and TCGA (**Figure 3C**) revealed positive correlation with cell division and replication, in line with previous results. Surprisingly, senescence was negatively correlated with estrogen response and UV response DN.

We next explored how senescence influence the tumor microenvironment (TME), employing ESTIMATE analysis for stromal and immune score, Xcell analysis for fibroblasts and immune cells, and ssGSEA analysis for ECM pathway activation, TGF-b signaling activation, angiogenesis score, immunogenic cell death and apoptosis. For the stromal compartment, CC2 had significantly suppressed stromal development in both TCGA (**Figure 3D**) and METABRIC (**Supplementary Figure 2B**). For the immune compartment, CC2 had lower immune score and lower infiltration of CD8^+^T cells and macrophage (**Figure 3E**, **Supplementary Figure 2C**). CC1 and CC2 had comparable TMB (**Supplementary Figures 2D, E**). As different forms of cell death have profound effects on the surrounding microenvironment, we determined that CC2 underwent less immunogenic cell death and apoptosis, potentially causing the dissimilar TME (**Figure 3E**, **Supplementary Figure 2C**). Interestingly, further analysis of paired whole-exon sequencing (WES) from TCGA and METABRIC revealed high mutation frequency of TP53 in CC2 (48%) but not CC1 (11%) (**Figure 3F**, **Supplementary Figure 2F**). Previous studies reported that the activated p53 modulates senescence with a dual effect of promoting or, in some cases, inhibiting the senescence program. Re-expression of P53^WT^ in the TP53 truncated cell line MDA-MB-436 breast cancer cell line significantly inhibited senescence, while P53^R175H^ slightly increased senescence compared to P53^null^ (**Figure 3G**, **Supplementary Figure 2G**). The results have been verified by transient re-expression of P53WT versus P53^R273H^ in the P53KO SW1990 cell line derived from human pancreatic cancer (**Supplementary Figure 2H**).

### Validation of senescence at the protein level

**Figure 4:**
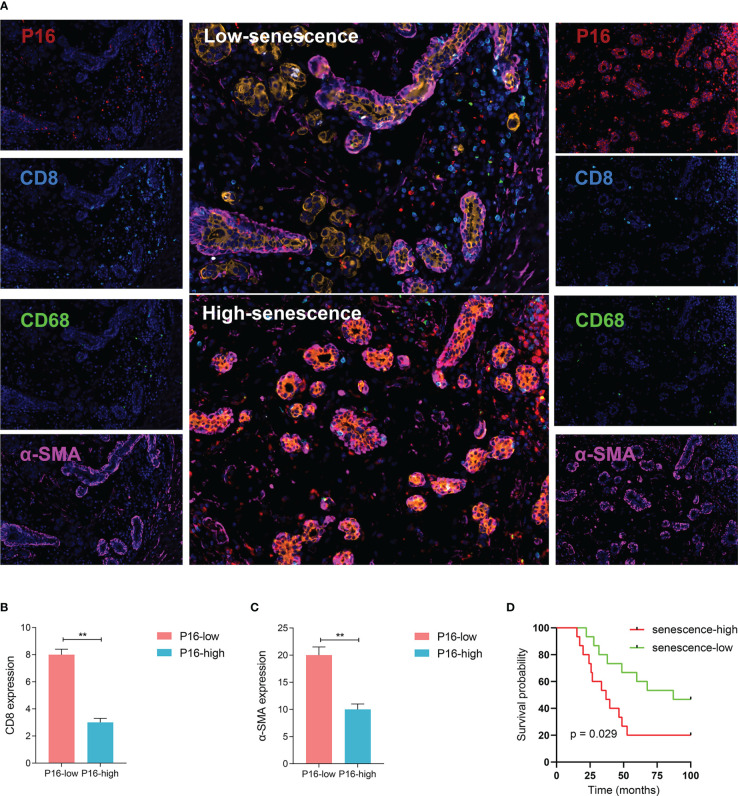
Visualized validation of the biological and clinical prognostic effects of senescence. **(A)** Representative multiplex immunohistochemistry (mIHC) images of patient samples with low-senescence (top panel) and high-senescence (bottom panel), and each color panel on the side. Markers were visualized by colors, with P16 in red, CD8 in turquoise, CD68 in green, a-SMA in pink, KI-67 in white and Pan-CK in orange (single color panels for a-SMA and KI-67 in supplementary), and DAPI counterstained. **(B, C)** Comparison of immune and stromal content from mIHC images between the high- and low-senescence groups, **(B)** defining immune status stromal status from the fluorescent intensity of CD8 in tumor region (annotated based on Pan-CK expression), and **(C)** similarily for stromal status and a-SMA fluorescence. **(D)** P16-based stratification of patients into senescence-high and senescence-low groups and comparison of overall survival by Kaplan-Meier curve. **p < 0.01.

To further validate the relationship between senescence and TME at translational level, a sophisticated method of multiplex immunofluorescence histochemistry, which allows simultaneous detection of multiple target proteins, was employed to analyze the protein expression of the five genes, PANCK, α-SMA, CD8, CD68, P16, identified above on a BRCA tissue microarray. The five proteins were the marker of epithelial cell, fibroblast, CD8^+^T cell, macrophage and senescence, respectively. High expression of P16 corresponds to low expression of PANCK, α-SMA, CD8, and CD68, suggesting low stromal development and low infiltration of immunocyte (**Figures 4A–C**).

To better understand the status of senescence and prognosis for BRCA patients, we included 30 BRCA patients in the National Cancer Center in China who have pathological tissue. According to the expression level of P16 determined by immunofluorescence, we divided the patients into high and low senescence groups by midline expression. Survival analysis showed higher OS in low senescence group (log-rank test, P = 0.029; median OS 87.0 months versus 36.9 months, **Figure 4D**).

### Construction of a senescence scoring model

**Figure 5:**
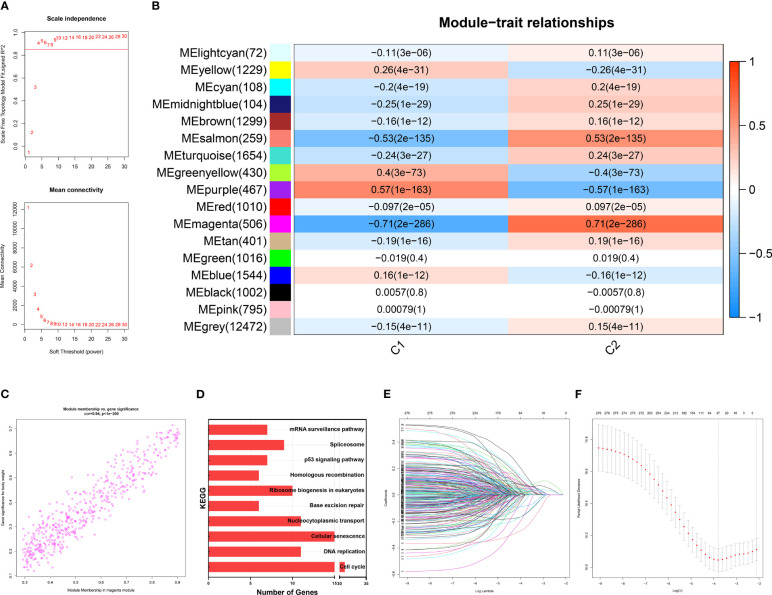
Identification of gene networks associated with CC1 and CC2 subtypes using WGCNA. **(A)** Analysis of the scale-free fit index for various soft-thresholding powers (β). 6 was considered the fittest power value. **(B)** Heatmap of correlations between gene modules and CC1 and CC2 subtypes. **(C)** Scatter plot depicting the correlation between gene significance and gene module membership in the magenta module. **(D)** Bar plot showing the significant enrichment results for genes in magenta module on KEGG pathways. **(E)** Different gene combinations and corresponding LASSO coefficients. **(F)** Parameter selection in the LASSO (least absolute shrinkage and selection operator) model. Independent Confidence intervals under each lambda were shown.

On a population-based view, senescence markedly affected the prognosis of BRCA patients. Therefore, we constructed a subtyping method, Sindex that allowed characterization of senescence status and subsequent outcome prediction for each patient. We first applied weighted gene co-expression network analysis (WGCNA) to all genes in the METABRIC dataset. After adjustments of WGCNA parameters, the starting genes were divided into 17 modules by average linkage hierarchical clustering (**Figures 5A, B**). The magenta module (506 genes) exhibited the highest correlation with CC2 (Pearson’s correlation coefficient = 0.71, P < 0.001) (**Figures 5B, C**), collectively termed CC2 or senescence-high related genes (SHRGs). Next, the 506 SHRGs were primarily screened by univariate Cox regression, leaving 278 OS-correlated SHRGs for further analysis (**Supplementary Table 5**). Enrichment analysis of the SHRGs demonstrated significant correlation with cellular senescence and cell division (**Figure 5D**). Finally, the LASSO regression algorithm screened out 37 genes for Sindex calculation (**Figures 5E, F**, **Supplementary Table 2**).

### Validation of Sindex to effectively predict patient outcomes based on senescence

**Figure 6:**
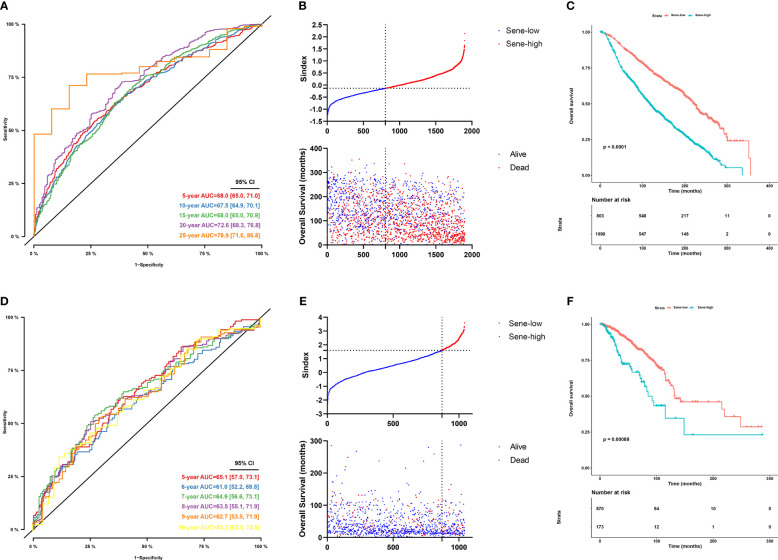
Evaluation of the performance of the senescence-based Sindex in METABRIC dataset and external validation in the TCGA-BRCA datasets. **(A)** Time-dependent ROC for 5-, 10 -,15-,20- and 25-year predictions of overall survival for the Sindex in the METABRIC dataset. **(B)** Relationship between the Sindex (upper), and the survival status of patients in different senescence subtype (bottom) in METABRIC dataset. **(C)** Kaplan-Meier survival curves of the Sindex. Patients from the METABRIC dataset are stratified into two groups according to the optimal cut-off values for the Sindex. **(D)** Time-dependent ROC for 5-, 10 -,15-,20- and 25-year predictions of overall survival for the Sindex in the TCGA-BRCA dataset. **(E)** Relationship between the Sindex (upper), and the survival status of patients in different senescence subtype (bottom) in TCGA-BRCA dataset. **(F)** Kaplan-Meier survival curves of the Sindex. Patients from the TCGA-BRCA dataset are stratified into two groups according to the optimal cut-off values for the Sindex.

Sindex was first internally validated with the METABRIC dataset, yielding an impressive predicting ability for OS (5-year AUC = 0.680, 10-year AUC = 0.675, 15-year AUC = 0.680, 20-year AUC = 0.726, 25-year AUC = 0.789) as indicated by the time-dependent ROC curve analysis (**Figure 6A**). External validation in the TCGA dataset further confirmed the robustness of Sindex in predicting OS for breast cancer patients (5-year AUC = 0.651, 10-year AUC = 0.632) (**Figure 6D**). The best range of OS prediction was 5-25 years, suggesting that Sindex was especially good for long-term prognosis, making it useful for BRCA, which is a chronic disease.

Looking at the Sindex of individual patients, an optimal cutoff value was selected with the maximally selected rank statistics from the ‘maxstat’ R package to define high senescence and low senescence subtypes, termed Sene-high and Sene-low respectively (**Figures 6B, E**). Kaplan-Meier survival analysis demonstrated clear separation of the two survival curves and shorter OS of the Sene-high subtype in METABRIC (log-rank test, P < 0.0001; median OS 19.6 months versus 56.2 months, **Figure 6C**) as well as TCGA (log-rank test, P < 0.001; median OS 19.6 months versus 56.2 months, **Figure 6F**).

**Figure 7:**
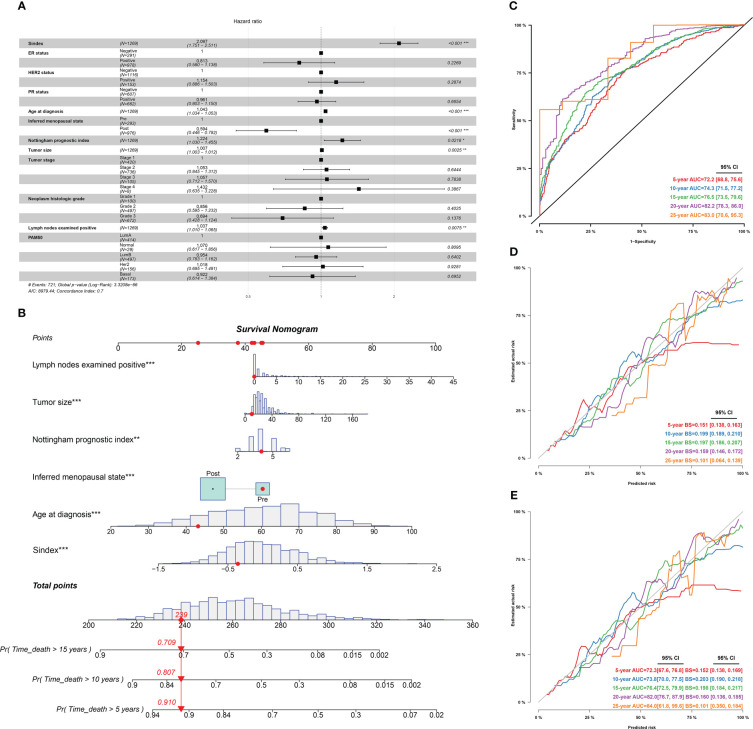
Identification of independent prognostic predictors and validation of the nomogram in predicting overall survival of breast cancer. **(A)** Forest plots show HR and p-values for Sindex and clinicopathological parameters based on multivariate COX regression. **(B)** A prognostic nomogram incorporating Sindex and clinical parameters predicting 5-, 10 - and 15-year overall survival of PDAC. **(C)** Time-dependent ROC for 5-, 10 -,15-,20- and 25-year predictions of overall survival for the nomogram in the METABRIC dataset. **(D)**. Calibration curves of the nomogram for predicting the probability of OS at 5-, 10 -,15-,20- and 25-year. **(E)** Internal validation of the nomogram using bootstrapping method *p < 0.05, **p < 0.01, ***p < 0.001.

Furthermore, we used univariant Cox regression to identify all OS-related characteristics with significant regression coefficients and p-values (**Supplementary Table 3**), and then used multivariate Cox regression to determine that Sindex is an independent and robust prognostic factor (HR, 2.097; 95% CI, 1.751–2.511; P < 0.001; **Figure 7A**). Based OS-related characteristics, we constructed a nomogram (**Figure 7B**) that could accurately predict the probability of 5-, 10-, and 15-year survival for BRCA patients (**Figures 7C–E**). The Sindex, lymph nodes positivity, tumor size, Nottingham prognostic index, inferred menopausal state, and age were incorporated into the nomogram as related predictors of breast cancer patient survival.

### Senescence and drug resistance

**Figure 8:**
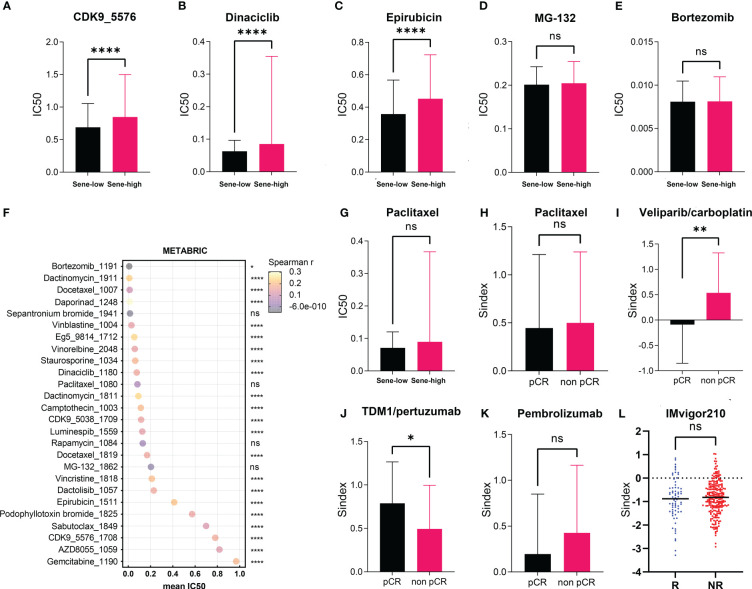
Exploration of Sindex for therapeutic response prediction. **(A–G)**. oncoPredict prediction of IC50 for different chemotherapeutic drugs in patients with BRCA from the TCGA dataset.**(A–F)** Comparison of predicted IC50 for **(A)** CDK9, **(B)** Dinaciclib, **(C)** Epirubicin, **(D)** MG-132, **(E)** Bortezomib, **(F)** Paclitaxel between sene-high and sene-low. **(G)** visualization of each drugs’ predicted IC50 and correlation with Sindex. **(H–K)** comparisons of Sindex between pCR and non-pCR patients in the ISPY-2 trial to **(H)** Paclitaxel, **(I)** Veliparib/carboplatin, **(J)** TDM1/pertuzumab and **(K)** Pembrolizumab. **(L)** comparison of Sindex between R and NR patients in the IMVigor210 trial to atezolizumab * p < 0.05, **p < 0.01, ****p < 0.0001. ns, no significance.

Using the Oncopredict algorithm for drug response prediction, we demonstrated that the Sene-high(subtype were resistant to CDK inhibitors (CDK9i-AZ5576 and Dinaciclib) (**Figures 8A, B**) and Epirubicine (**Figure 8C**), but there was no significant difference in sensitivity to proteasome inhibitors (MG132 and Bortezomib) between Sene-low and Sene-high (**Figures 8D, E**). Further analysis revealed that Bortezomib had the lowest IC50 and the most negative correlation with Sindex (**Figure 8F**), suggesting that Bortezomib may be the most effective drug for the group of high-senescence and high-risk BRCA patients. Interestingly, the Sene-high subtype was resistant to some anti-microtubule chemotherapeutic drugs (**Supplementary Figures 3A, B**), but there was no significant difference in paclitaxel sensitivity between the two groups, suggesting more complex effect-response-resistance mechanisms of these drugs (**Figure 8G**).

We further explored the real-world effect of senescence on drug response in transcriptomic data from I-SPY2, a clinical research of neoadjuvant treatment for BRCA patients. There was no significant difference in Sindex bewtween pCR and non-pCR patients receiving paclitaxel, in line with previous result (**Figures 8G, H**). However, the significantly higher Sindex in non-pCR patients receiving veliparib and carboplatin suggested resistance to this treatment strategy conferred by senescence (**Figure 8I**). Therapeutic response to Neratinib (TKI), MK2206 (AKT inhibitor), Ganitumab (anti-IGF1R-mAb), and Trebananib (neutralizing peptide against Ang1/2) are not significantly affected by senescence, but non pCR patients to the Hsp90 inhibitor Ganetespib have significantly higher Sindex (**Supplementary Figures 4A, B, D–F**). As to patients receiving TDM1 and pertuzumab, pCR patients had significantly higher Sindex (**Figure 8J**), but patients with pCR and non-pCR to pertuzumab had comparable Sindex (**Supplementary Figure 4C**), suggesting that the high-senescence and high-risk group of patients may benefit from T-DM1 treatment. We noted that high-senescence patients may not benefit from Pembrolizumab immunotherapy (**Figure 8K**), consistent with previous results suggesting lower immune infiltration into senescent tumors. This was further confirmed by data from urothelial cancer patients treated with Atezolizumab in IMvigor 210 (**Figure 8L**).

## Discussion

In 2020, the incidence of BRCA reached 11.7%, surpassing lung cancer for the first time to become the most diagnosed cancer in the world (1). Despite widely used classification system by molecular markers, the heterogeneous nature of BRCA poses difficulty onto prognosis and therapeutic decision (2, 44). Therefore, exploring new methods of BRCA subtyping has become the most urgent task in bringing precision medicine into reality (45). In this study, we identified senescence as a hallmark of BRCA that is associated with distinctive TME and genomic alterations. Based on the senescent characteristics of BRCA, we developed a subtyping scheme Sindex that provides an insightful perspective on the biological property of tumors from a new perspective.

We detected that high senescence is associated with significantly worse survival outcome in BRCA patients, suggesting that the activation of cellular senescence pathways may promote malignancy and therapeutic resistance. Although senescence is often considered a defense mechanism against cancer due to its cell cycle arresting properties (46), senescence is a potentially reversible process of epigenetic and transcriptional changes, and senescent dormancy may offer protection by immune-evasion, drug resistance, and resistance to apoptosis or other forms of cell death (47). Sun et al. reported that senescence is associated with good survival in gastric cancer (48), suggesting multimodal function and different mechanisms of senescence in different cancers.

The complexity of senescence deserves further investigation on the potential biological effect of senescence in BRCA. Different methods of data analysis in two datasets both reached the same conclusions: (1) senescence is associated with extensive cell replication and suppression of stroma and immune development; (2) high senescence suppresses stromal development and immune infiltration in the TME. SASP is a well-investigated property of senescence generally considered to be pro-tumorigenic, as senescent tumor cells have specific secretory phenotypes marked by increased secretion of pro-inflammatory (but not anti-tumor immunity), bioactive molecules including IL-6, IL-1a, chemokines (CXCL8), VEGF, and proteases (49). However, our result did not reveal a significant difference in SASP pathway activity between the Sene-high and the Sene-low groups, suggesting other mechanisms by which senescence remodels the TME. We further investigated other key characteristics crucial for anti-tumoral immunity, as cell death and antigen release are crucial for the initiation of anti-tumoral immune response, we indeed found out that senescence suppresses immunogenic cell death, resulting in lower antigen-presenting cell infiltration.

To increase the clinical application value and create better clinical practicability, we successfully constructed a novel senescence-related scoring tool (Sindex) to determine the prognostic risk of BRCA. The Sindex effectively stratified patients with BRCA into high- and low-risk groups. Survival analysis revealed that the Sene-high group had shorter OS than Sene-low group, and ROC curves exhibited a great predictive capacity of Sindex for the 5-, 10-, 15-, 20- and 25-year survival of BRCA. The model is validated in two individual datasets, METABRIC and TCGA.

Previous studies have demonstrated that cellular senescence could increase the drug resistance and side effects of the chemotherapy (50–52). Using the Oncopredict algorithm and I-SPY2 dataset, we found out that indeed, most drugs have a higher IC50 value in the Sene-high group, especially CDK inhibitors, epirubicin, and olaparib. Such resistance is explainable as those drugs share common mechanisms in inhibition of cell cycle progression through cell death induced damage. On the other hand, patients with high senescence may benefit from metabolic drugs such as proteosome inhibitors and MTORC inhibitors, consistent with the fact that senescent cells are metabolically active. Using the I-SPY2 and IMvigor 210 data sets, we noted that high-senescence patients may not benefit from immune checkpoint therapies, consistent with previous results showing lower immune infiltration in Sene-high subtype.

Our study has the limitation that the high-throughput sequencing data sets for initial analysis were relatively insufficient as it was simply obtained from public databases. The corresponding results and conclusions remain to be further investigated through more external congeneric research and should be validated *via* functional experiments *in vivo* and *in vitro*. Furthermore, several conclusions of this study require further research to confirm its reproducibility, improve the clinical application of senescence-related clusters, and elaborate on the role of Sindex in predicting the response to cancer therapy for BRCA.

## Conclusions

In conclusion, our study lead to the identification of senescence subtypes that are associated with significantly different TME and survival outcomes. Our analysis demonstrated that the Sindex can be used to identify not only patients at different risk levels for the OS but also patients who would benefit from some cancer therapeutic drugs. Nevertheless, the validation of our findings in a wide spectrum of patient cohorts, and the findings that the senescence features reflect biological and clinical characteristics associated with sensitivity or resistance to the therapy, would pave a way for developing more rational therapy recommendations and promoting personalized cancer therapy.

## Data availability statement

The original contributions presented in the study are included in the article/**Supplementary Material**, further inquiries can be directed to the corresponding authors.

## Ethics statement

Written informed consent was obtained from the individual(s) for the publication of any potentially identifiable images or data included in this article.

## Author contributions

JZ, JH, FM, and BX designed the study and revised the manuscript. JZ and JH performed the data analysis and wrote the manuscript with inputs from CL. Authors CL and DL conducted the multiplex immunofluorescence immunohistochemistry and imaging. JZ, JH, CL, and DL did data collection. All authors have read and approved the final manuscript. All authors contributed to the article and approved the submitted version.

## Supplementary material

Supplementary Figure 1 Characterization of patient features associated with senescence-based consensus clustering. **(A)** Correlation analysis between age and ssGSEA of BRCA-SRGs in TCGA revealed no significant correlation (P = 0.281). **(B)** Comparison of PAM50 subtypes between CC1 and CC2 in TCGA revealed differential enrichment of PAM50 subtypes associated with senescence status. **(C)** Consensus clustering matrix based on BRCA-SRGs with k=2 in the METABRIC cohort. **(D)** Heatmap showing differences in ssGSEA scores for each senescence-related gene set between CC1 and CC2 subtypes, along with clinicopathological features. **(E)** Correlation analysis between age and ssGSEA of BRCA-SRGs in METABRIC revealed no significant correlation (p=0.129). **(F)** Comparison of PAM50 subtypes between CC1 and CC2 in METABRIC revealed differential enrichment of PAM50 subtypes associated with senescence statuses. **(G)** Principal component analysis of expression profiles to differentiate the two subtypes in the METABRIC cohort, with CC1 marked in green and CC2 marked in red.

Supplementary Figure 2 Validation of the biological impacts of senescence. **(A)** GSEA enrichment showing upregulated pathways in CC2 versus CC1 in METABRIC. **(B, C)** Comparison of stromal **(B)** and immune **(C)** condition between CC1 and CC2 in METABRIC. **(D, E)** Comparison of tumor mutation burden (TMB) between CC1 and CC2 in TCGA **(D)** and METABRIC **(E)**. **(F)** Comparison of mutation frequencies between CC1 and CC2 in the METABRIC dataset. **(G)** The effect of wildtype P53 (P53WT) and mutant P53 (P53MUT) re-expression on senescence in terms of p16, p21, Rb, IL-6, IL-1α, ICAM-1 and TGF-β mRNA content measure by RT-qPCR in the P53 truncated cell line MDA-MB-436. **(H)** The effect of wildtype P53 (P53WT) and mutant P53 (P53MUT) re-expression on senescence in terms of p16 and p21 mRNA content measure by RT-qPCR in the P53 knocked out cell line SW1990.

Supplementary Figure 3 OncoPredict prediction of IC50 for different chemotherapeutic drugs in patients with BRCA from the METABRIC **(A)** and TCGA **(B)** dataset.

Supplementary Figure 4 Correlation of Sindex with therapeutic responses in real-world data from I-SPY2 clinical trial by comparision of Sindex between responders and non-responders to **(A)** Neratinib, **(B)** MK2206, **(C)** Pertuzumab, **(D)** Ganitumab, **(E)** Ganetespid, **(F)** Trebananib

Supplementary Table 1 Summary of senescence related gene sets and genes

Supplementary Table 2 278 OS-correlated senescence-high related genes and 37 Sindex genes

Supplementary Table 3 OS-related clinical characteristics

Supplementary Table 4 Details of the datasets included in this study

Supplementary Table 5 Information of patients included in this study
