# Supplementary figures and images for: Tumor senescence leads to poor survival and therapeutic resistance in human breast cancer
Source: Front Oncol. 2023 Mar 2;13:1097513. doi: 10.3389/fonc.2023.1097513 (PMC10019818; doi:10.3389/fonc.2023.1097513)

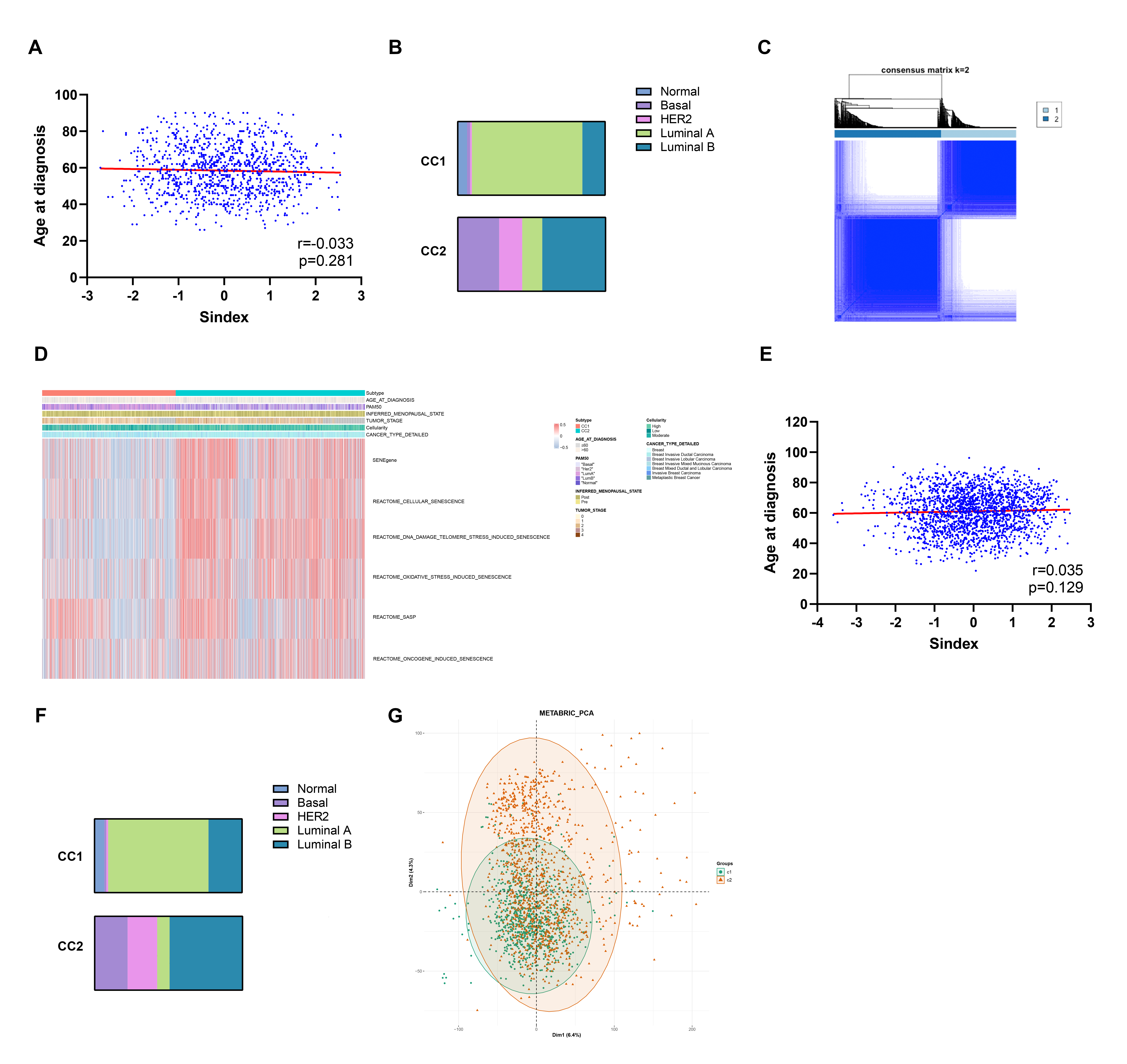

Supplement: Supplementary Figure 1 — Characterization of patient features associated with senescence-based consensus clustering. (A) Correlation analysis between age and ssGSEA of BRCA-SRGs in TCGA revealed no significant correlation (P = 0.281). (B) Comparison of PAM50 subtypes between CC1 and CC2 in TCGA revealed differential enrichment of PAM50 subtypes associated with senescence status. (C) Consensus clustering matrix based on BRCA-SRGs with k=2 in the METABRIC cohort. (D) Heatmap showing differences in ssGSEA scores for each senescence-related gene set between CC1 and CC2 subtypes, along with clinicopathological features. (E) Correlation analysis between age and ssGSEA of BRCA-SRGs in METABRIC revealed no significant correlation (p=0.129). (F) Comparison of PAM50 subtypes between CC1 and CC2 in METABRIC revealed differential enrichment of PAM50 subtypes associated with senescence statuses. (G) Principal component analysis of expression profiles to differentiate the two subtypes in the METABRIC cohort, with CC1 marked in green and CC2 marked in red. [file Image_1.tif]

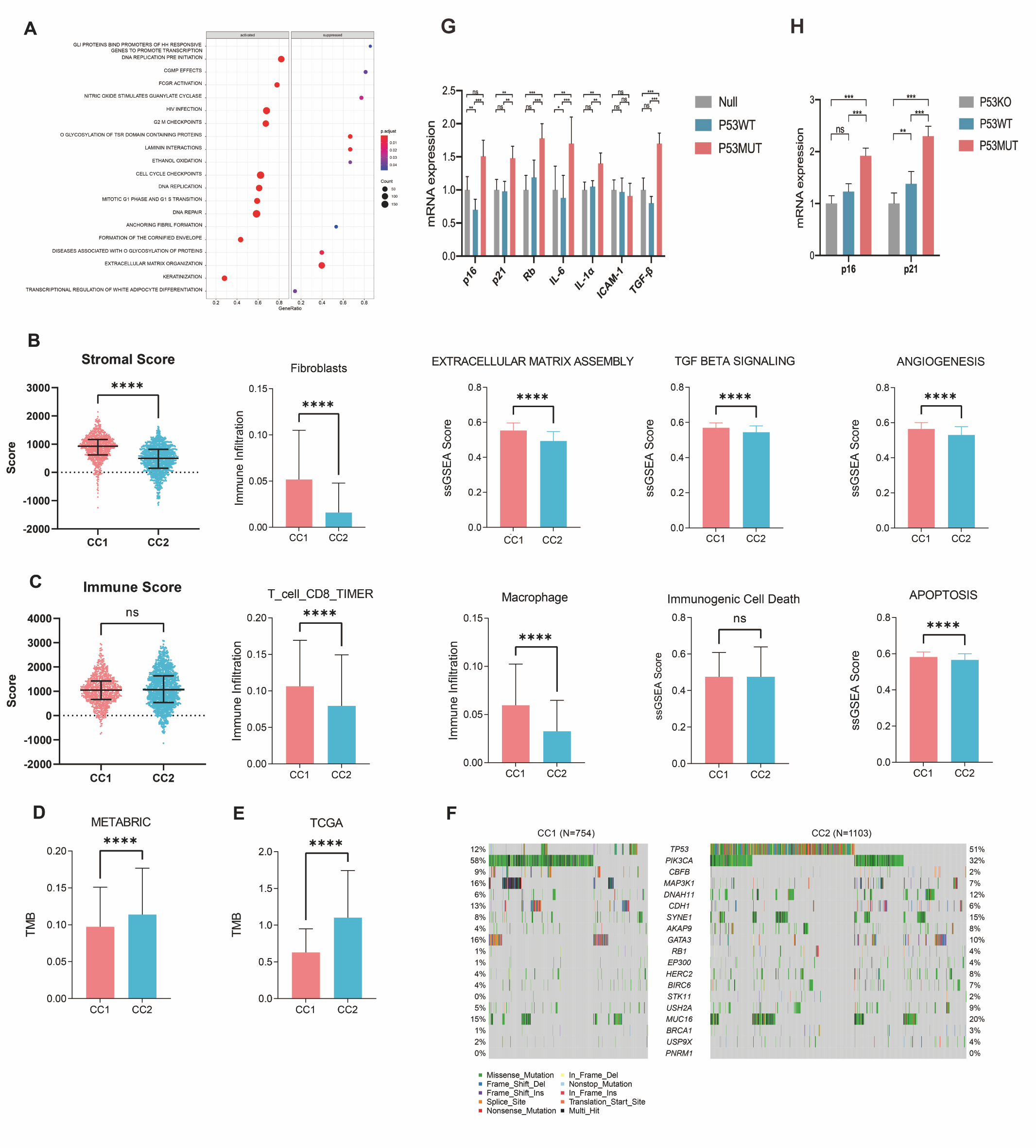

Supplement: Supplementary Figure 2 — Validation of the biological impacts of senescence. (A) GSEA enrichment showing upregulated pathways in CC2 versus CC1 in METABRIC. (B, C) Comparison of stromal (B) and immune (C) condition between CC1 and CC2 in METABRIC. (D, E) Comparison of tumor mutation burden (TMB) between CC1 and CC2 in TCGA (D) and METABRIC (E). (F) Comparison of mutation frequencies between CC1 and CC2 in the METABRIC dataset. (G) The effect of wildtype P53 (P53WT) and mutant P53 (P53MUT) re-expression on senescence in terms of p16, p21, Rb, IL-6, IL-1α, ICAM-1 and TGF-β mRNA content measure by RT-qPCR in the P53 truncated cell line MDA-MB-436. (H) The effect of wildtype P53 (P53WT) and mutant P53 (P53MUT) re-expression on senescence in terms of p16 and p21 mRNA content measure by RT-qPCR in the P53 knocked out cell line SW1990. [file Image_2.tif]

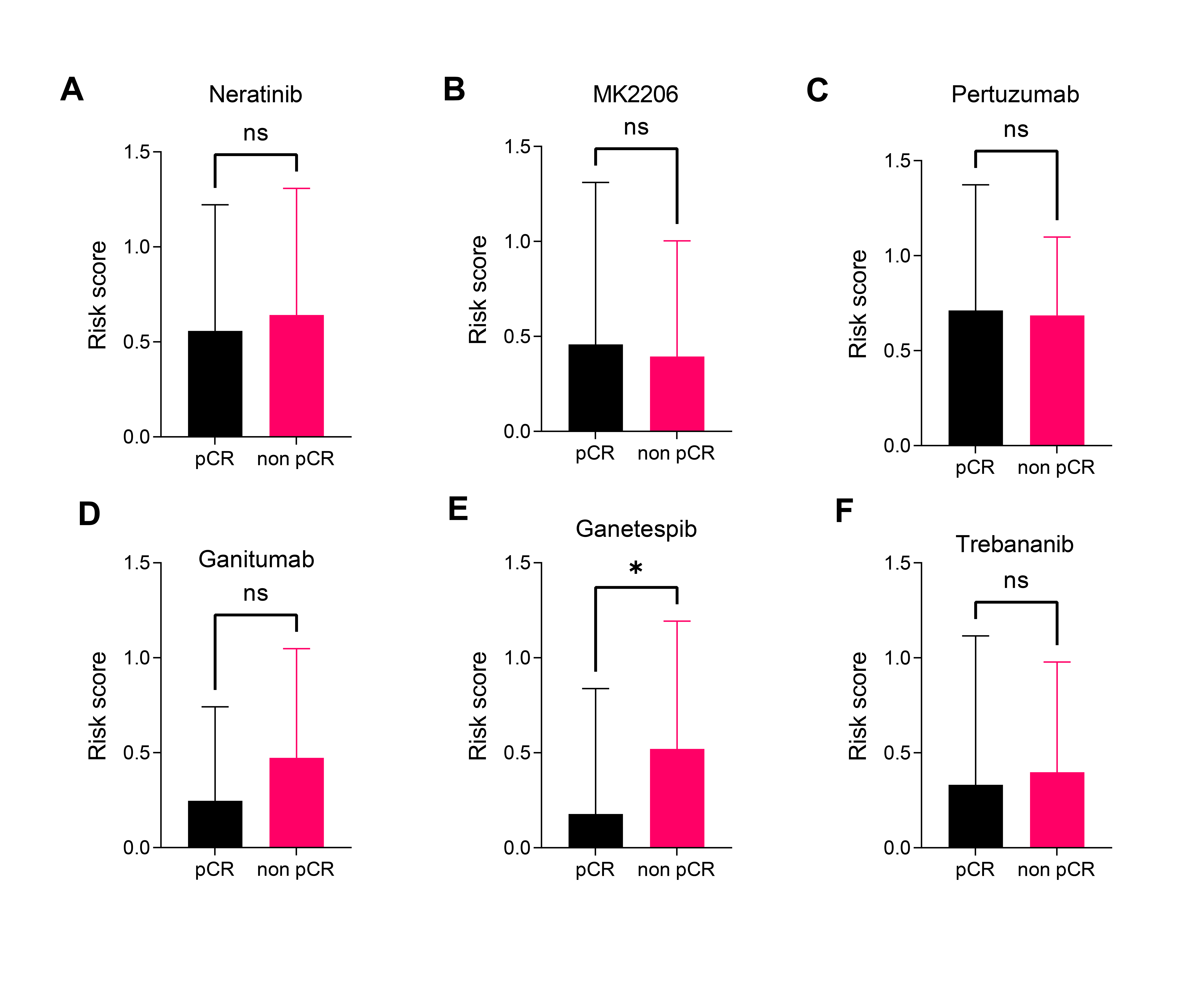

Supplement: Supplementary Figure 4 — Correlation of Sindex with therapeutic responses in real-world data from I-SPY2 clinical trial by comparision of Sindex between responders and non-responders to (A) Neratinib, (B) MK2206, (C) Pertuzumab, (D) Ganitumab, (E) Ganetespid, (F) Trebananib [file Image_4.tif]
